# Rural and socioeconomic differences in the effectiveness of the HEART Pathway accelerated diagnostic protocol

**DOI:** 10.1111/acem.14643

**Published:** 2023-01-03

**Authors:** James C. O'Neill, Nicklaus P. Ashburn, Brennan E. Paradee, Anna C. Snavely, Jason P. Stopyra, Greg Noe, Simon A. Mahler

**Affiliations:** ^1^ Department of Emergency Medicine Wake Forest School of Medicine Winston‐Salem North Carolina USA; ^2^ Section on Cardiovascular Medicine, Department of Internal Medicine Wake Forest School of Medicine Winston‐Salem North Carolina USA; ^3^ Department of Biostatistics and Data Science Wake Forest School of Medicine Winston‐Salem North Carolina USA; ^4^ Department of Implementation Science Wake Forest School of Medicine Winston‐Salem North Carolina USA; ^5^ Department of Epidemiology and Prevention Wake Forest School of Medicine Winston‐Salem North Carolina USA

## Abstract

**Background:**

The HEART Pathway is a validated accelerated diagnostic protocol (ADP) for patients with possible acute coronary syndrome (ACS). This study aimed to compare the safety and effectiveness of the HEART Pathway based on patient rurality (rural vs. urban) or socioeconomic status (SES).

**Methods:**

We performed a preplanned subgroup analysis of the HEART Pathway Implementation Study. The primary outcomes were death or myocardial infarction (MI) and hospitalization at 30 days. Proportions were compared by SES and rurality with Fisher's exact tests. Logistic regression evaluated for interactions of ADP implementation with SES or rurality and changes in outcomes within subgroups.

**Results:**

Among 7245 patients with rurality and SES data, 39.9% (2887/7245) were rural and 22.2% were low SES (1607/7245). The HEART Pathway identified patients as low risk in 32.2% (818/2540) of urban versus 28.1% (425/1512) of rural patients (*p* = 0.007) and 34.0% (311/915) of low SES versus 29.7% (932/3137) high SES patients (*p* = 0.02). Among low‐risk patients, 30‐day death or MI occurred in 0.6% (5/818) of urban versus 0.2% (1/425) rural (*p* = 0.67) and 0.6% (2/311) with low SES versus 0.4% (4/932) high SES (*p* = 0.64). Following implementation, 30‐day hospitalization was reduced by 7.7% in urban patients (adjusted odds ratio [aOR] 0.76, 95% confidence interval [CI] 0.66–0.87), 10.6% in low SES patients (aOR 0.68, 95% CI 0.54–0.86), and 4.5% in high SES patients (aOR 0.83, 95% CI 0.73–0.94). However, rural patients had a nonsignificant 3.3% reduction in hospitalizations.

**Conclusions:**

HEART Pathway implementation decreased 30‐day hospitalizations regardless of SES and for urban patients but not rural patients. The 30‐day death or MI rate was similar among low‐risk patients.

## INTRODUCTION

Emergency departments (ED) in the United States see 7–9 million patients with acute chest pain annually.^1^, ^2^ Care of these patients is focused on accurate diagnosis of acute coronary syndrome (ACS) and costs an estimated 10 billion dollars annually.^3^, ^4^, ^5^, ^6^, ^7^ However, these costly and lengthy evaluations ultimately diagnose fewer than 10% with ACS. To address these inefficiencies, accelerated diagnostic protocols (ADPs), such as the history, electrocardiogram (ECG), age, risk factors, and troponin pathway (HEART Pathway), have been developed to improve ACS risk stratification and lower costs.^8^, ^9^, ^10^, ^11^

The HEART Pathway is a validated and widely used ADP for the risk stratification of ED patients with chest pain.^12^, ^13^, ^14^ In prior studies, the HEART Pathway safely increased early discharges from the ED, decreased cardiac testing, hospital length of stay, and costs.^14^, ^15^, ^16^ Thus, its safety and effectiveness have been well established, including in important subgroups such as women and African Americans. However, its performance has yet to be tested among rural and low socioeconomic status (SES) patients.

Patients living in rural areas and those living with poverty have significant cardiovascular health disparities compared to their urban and high SES counterparts.^17^, ^18^, ^19^, ^20^ Since the 1980s, patients in rural areas have suffered what has been called the “rural mortality penalty,” with increased morbidity and mortality from the same illnesses as their urban counterparts.^21^, ^22^, ^23^ The American Heart Association published a call to action to systematically reduce rural health care disparities.^24^ Lower SES is also associated with decreased life expectancy and higher rates of ACS.^25^, ^26^ Patients with low SES are more likely to experience a cardiovascular event and have worse outcomes than their high SES counterparts.^27^, ^28^ Given these well‐known disparities, we seek to determine whether the safety and effectiveness of the HEART Pathway differs among rural versus urban patients and for patients with low versus high SES.

## METHODS

### Study design and oversight

This study was a preplanned secondary analysis of the HEART Pathway Implementation Study. Participants were recruited from November 2013 to January 2016 after approval by the Wake Forest University Health Sciences Institutional Review Board. The trial was registered with clincaltrials.gov (NCT02056964). The HEART Pathway Implementation Study's methods have been previously described.^29^ This article follows the Strengthening and Reporting of Observational Studies in Epidemiology (STROBE) guidelines.^30^

### Study setting and population

The study was conducted at three hospitals in North Carolina: one large urban academic medical center with approximately 114,000 ED visits annually; a freestanding ED in a rural county with approximately 12,000 annual ED visits; and a small, rural county community hospital with approximately 37,000 annual ED visits. The study population included patients ≥21 years of age being investigated for possible ACS. Patients with ST‐segment elevation myocardial infarction (STEMI) were excluded. Patients without rurality and SES data were excluded from this analysis. A patient flow diagram is included as Figure S1.

At the academic medical center and freestanding ED, participants were accrued into the preimplementation (November 2013–October 2014) or postimplementation (February 2015–January 2016) cohort. The community ED accrued patients in the preimplementation cohort from January 2015–July 2015 and the postimplementation cohort from August 2015–January 2016. Patients were accrued into their cohort based on their first ED visit for possible ACS. To prevent repeat ED users/high ED utilizers from being overly represented in the preimplementation cohorts, patients with an ED visit for possible ACS at each site the year before the study were excluded (*N* = 523). For transfers, patients' care at the receiving hospital was considered part of their index encounter.

### Data collection

Index encounter data were extracted from the electronic health record (EHR; Clarity‐Epic Systems Corporation). EHR variables or diagnoses and procedure codes (CPT, ICD‐9, and ICD‐10) were used to obtain patient demographics, comorbidities, troponin results, HEART Pathway assessments, dispositions, diagnoses, and vital status. For 30‐day outcomes, data were collected from the EHR, insurers' claims data, and state death index data. Patients using Blue Cross Blue Shield (the dominant insurer in North Carolina), MedCost, and North Carolina Medicaid had claims information available for study use. Death index data from the North Carolina State Center for Health Statistics was used.

### HEART Pathway implementation

The HEART Pathway ADP was fully integrated into EPIC as an interactive clinical decision support (CDS) tool. All adult patients presenting with chest pain who had at least one troponin ordered during the postimplementation period had an interruptive pop‐up alert for the HEART Pathway CDS in the EHR. In addition, the HEART Pathway tool was integrated into an EHR flowsheet, which provided manual access to the HEART Pathway for patients presenting with other symptoms concerning for ACS, such as dyspnea or arm pain.

The HEART Pathway CDS tool prompted providers to answer a series of questions to prospectively risk stratify patients in real‐time. Patients with known coronary artery disease (CAD; prior MI, prior coronary revascularization, or coronary stenosis ≥ 70%) or acute ischemic ECG changes (e.g., new T‐wave inversions or ST‐segment depression in contiguous leads) were immediately classified as non–low risk.

**FIGURE 1:**
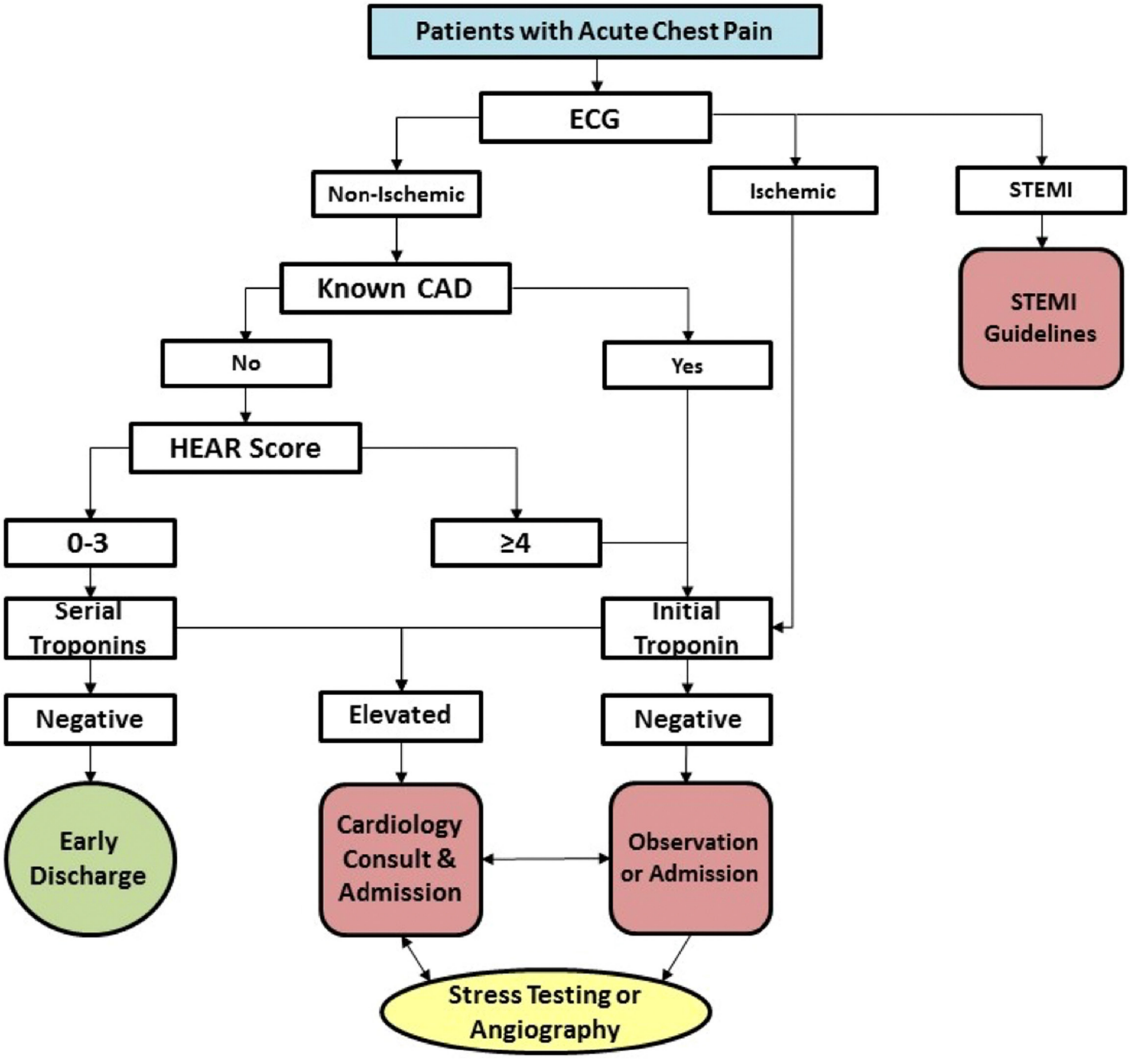
The HEART Pathway algorithm. CAD, coronary artery disease; STEMI, ST‐segment elevation myocardial infarction

Among patients without STEMI, known CAD, or acute ischemic ECG changes, providers answered additional flowsheet questions to determine a history, ECG, age, and risk factor score (HEAR score), calculated based on the HEART Pathway trial algorithm (Impathiq Inc.).^14^ The HEART Pathway risk assessment was automatically calculated based on the HEAR score and 0‐ and 3‐h troponin measures. Patients with HEAR scores ≤ 3 and without elevated troponin measures were classified as low risk and recommended for discharge without stress testing or coronary imaging. During the postimplementation period, discharged low‐risk patients were asked to follow up with their primary care physician within 30 days. Patients with a HEAR score ≥ 4, an elevated troponin, known CAD, or ischemic ECG changes were classified as non–low risk and designated for further testing (Figure 1). During the preimplementation period, the HEART Pathway CDS tool was not available to providers and HEAR scores were not recorded. Serum troponin was measured throughout the study period using the ADVIA Centaur platform TnI‐Ultra assay (Siemens) or the Access AccuTnI+3 assay (Beckman Coulter). The package insert 99th percentile upper reference limit for each assay was used to determine a positive result at 0 and 3 h.

### Rurality and SES

Consistent with prior studies, rurality (urban vs. rural location) and high versus low SES were determined using patient ZIP codes and geographic identifiers (GEOIDs). Uniform Data System (UDS) crosswalk files from 2010 were used to link a patient's ZIP code to the corresponding ZIP Code Tabulation Area 5‐Digit (ZCTA5). Each ZCTA5 was classified as either urban or rural based on census defined urbanized areas (UAs) and urban clusters (UCs). If at least 50% of the population in a ZCTA5 lived in a UA or UC then the ZCTA5 was classified as urban; otherwise it was classified as rural.^31^, ^32^, ^33^, ^34^, ^35^, ^36^ SES was determined using national area deprivation index (ADI) percentiles from the University of Wisconsin School of Medicine and Public Health.^37^ The ADI is a validated, factor‐based deprivation index that uses 17 poverty, education, housing, and employment indicators drawn from U.S. Census data to measure the SES of a census tract.^19^, ^20^, ^38^, ^39^, ^40^ GEOIDs from the U.S Census Bureau were linked to ADI percentiles when available. For patients who were missing a GEOID, ZIP+4 codes were linked to ADI percentile. Consistent with previous research, an ADI percentile at or above 85 was classified as low SES and below 85 was high SES.^39^, ^41^, ^42^

### Outcomes

The primary safety outcome was the composite of all‐cause death or MI at 30 days (inclusive of index visit). Coronary revascularization, a secondary endpoint, was defined as coronary artery bypass grafting or percutaneous coronary intervention. MI and coronary revascularization were determined using diagnosis and procedure codes validated by prior cardiovascular trials.^14^, ^16^, ^43^, ^44^, ^45^, ^46^ Major adverse cardiac events, the composite of all‐cause death, MI, or coronary revascularization, was also evaluated as a secondary endpoint.

The primary effectiveness outcome was hospitalization at 30 days (from index visit through 30‐day follow‐up). Hospitalization was defined as an inpatient admission, transfer, or observation stay (including index observation unit care). Secondary outcomes included objective cardiac testing (OCT; stress testing, coronary computed tomography angiography, or invasive coronary angiography) at index and through 30 days of follow‐up as well as early discharge rates, defined as the proportion of patients discharged from the ED without OCT. In the postimplementation period, nonadherence to the HEART Pathway was also determined, where nonadherence was defined as low‐risk patients receiving OCT or hospitalization or non–low‐risk patients receiving early discharge from the ED.

### Statistical analysis

Statistical design for the HEART Pathway Implementation Study is described previously.^13^, ^14^ Patient characteristics were described by pre‐ and postimplementation cohorts within each subgroup. Rurality, SES, and 30‐day death or MI proportions were compared between pre‐ and postimplementation cohorts using chi‐square tests. Postimplementation, the percentage of patients identified as low risk and non–low risk were calculated to determine the sensitivity, specificity, and positive and negative predictive of the HEART Pathway for death and MI. Corresponding 95% exact binomial confidence intervals (CI) were computed. For positive and negative likelihood ratios, 95% CIs were calculated using the method of Simel et al.^47^ The proportion of patients classified as low risk was compared between subgroups using Fisher's exact test. Nonadherence to the protocol was compared between subgroups using a chi‐square test.

Unadjusted logistic regression and absolute percentage differences were used to evaluate the relationship between pre‐ and postimplementation periods and study outcomes within each subgroup. Multivariable logistic regression models were then used to adjust for potential confounders, which were selected a priori: age, sex, race, insurance status, enrollment site, smoking, body mass index (BMI), rurality (for SES subgroups only), SES (for rurality subgroups only), and presence of chest pain versus other symptoms concerning for ACS (EHR flowsheet use). To test for significant differences in implementation between rurality and SES, logistic regression models were fit using the overall population including rurality by implementation cohort (pre vs. post) or SES by implementation cohort interaction terms. The same potential confounders were included in these models. Absolute percentage differences with 95% CIs were computed between subgroups for hospitalization, OCT, and early discharge in the pre‐ and postimplementation cohorts separately.

Patients were excluded from all analyses if they were missing GEOID or ZIP code as this was required to classify patients based on rurality and SES. Under the assumption that these data were missing at random, the complete case analysis (with adjustment) should provide unbiased estimates. This assumption is reasonable given that missing data were due to addresses being incomplete or erroneous or there being a mismatch between address, city, or state. Patients with missing HEAR scores were not excluded from any pre‐ versus postimplementation analyses.

## RESULTS

The HEART Pathway Implementation Study accrued 8474 patients over 24 months, of which 7245 had complete rurality and SES data (Figure 1). The cohort was 53.7% (3889/7245) female and 33.9% (2454/7245) non‐White with a median (IQR) age of 55 (45–66) years. There was a higher proportion of rural patients in the preimplementation cohort compared to the postimplementation cohort (43.1% [1375/3193] vs. 37.3% [1512/4052]; *p* < 0.001). The pre‐ and postimplementation cohorts had similar proportions of patients with low SES (21.7% [692/3193] vs. 22.6% [915/4052]; *p* = 0.36). The rate of 30‐day death or MI was similar pre‐ versus postimplementation (7.0% [222/3193] vs. 7.6% [309/4052]; *p* = 0.28). Cohort characteristics by rurality and SES are summarized in Table 1. Test characteristics of the HEART Pathway by subgroup are summarized in Tables 2 and 3. Safety and effectiveness endpoints by subgroup are summarized in Tables 4 and 5. Absolute percentage reductions for each subgroup are shown in Table 6. Safety events among low‐risk patients are summarized in Table S1.

**TABLE 1:** Pre‐ and postimplementation cohort characteristics by rurality

	Urban			Rural		
Patient characteristics	Total (*N* = 4358)	Pre (*n* = 1818)	Post (*n* = 2540)	Total (*N* = 2887)	Pre (*n* = 1375)	Post (*n* = 1512)
Age (years), median (IQR) years	54 (20)	54 (20)	54 (21)	55 (22)	55 (22)	55 (22)
Female	2388.0 (54.8)	975.0 (53.6)	1413.0 (55.6)	1501.0 (52.0)	719.0 (52.3)	782.0 (51.7)
Race						
White	2253.0 (51.7)	927.0 (51.0)	1326.0 (52.2)	2538.0 (87.9)	1212.0 (88.1)	1326.0 (87.7)
Non‐White	2105.0 (48.3)	891.0 (49.0)	1214.0 (47.8)	349.0 (12.1)	163.0 (11.9)	186.0 (12.3)
Ethnicity						
Hispanic or Latino	241.0 (5.5)	90.0 (5.0)	151.0 (5.9)	68.0 (2.4)	26.0 (1.9)	42.0 (2.8)
Low SES	1259.0 (28.9)	530.0 (29.2)	729.0 (28.7)	348.0 (12.1)	162.0 (11.8)	186.0 (12.3)
Site						
Academic	4084.0 (93.7)	1705.0 (93.8)	2379.0 (93.7)	1413.0 (48.9)	650.0 (47.3)	763.0 (50.5)
Freestanding	166.0 (3.8)	69.0 (3.8)	97.0 (3.8)	635.0 (22.0)	279.0 (20.3)	356.0 (23.5)
Community	108.0 (2.5)	44.0 (2.4)	64.0 (2.5)	839.0 (29.1)	446.0 (32.4)	393.0 (26.0)
Insurance status						
Private	1150.0 (26.4)	489.0 (26.9)	661.0 (26.0)	853.0 (29.5)	401.0 (29.2)	452.0 (29.9)
Public	2037.0 (46.7)	795.0 (43.7)	1242.0 (48.9)	1350.0 (46.8)	626.0 (45.5)	724.0 (47.9)
Other	1171.0 (26.9)	534.0 (29.4)	637.0 (25.1)	684.0 (23.7)	348.0 (25.3)	336.0 (22.2)
Risk factors						
Prior CAD	1053.0 (24.2)	444.0 (24.4)	609.0 (24.0)	815.0 (28.2)	383.0 (27.9)	432.0 (28.6)
Diabetes	1219.0 (28.0)	504.0 (27.7)	715.0 (28.1)	768.0 (26.6)	389.0 (28.3)	379.0 (25.1)
Hyperlipidemia	1827.0 (41.9)	748.0 (41.1)	1079.0 (42.5)	1235.0 (42.8)	581.0 (42.3)	654.0 (43.3)
Hypertension	2827.0 (64.9)	1205.0 (66.3)	1622.0 (63.9)	1797.0 (62.2)	872.0 (63.4)	925.0 (61.2)
Smoking	2584.0 (59.3)	1095.0 (60.2)	1489.0 (58.6)	1845.0 (63.9)	904.0 (65.7)	941.0 (62.2)
BMI ≥ 30 mg/kg^2^	1996.0 (47.2)	820.0 (46.0)	1176.0 (48.0)	1318.0 (47.0)	632.0 (47.1)	686.0 (46.9)
Cerebrovascular disease	562.0 (12.9)	227.0 (12.5)	335.0 (13.2)	349.0 (12.1)	171.0 (12.4)	178.0 (11.8)
Peripheral vascular disease	584.0 (13.4)	225.0 (12.4)	359.0 (14.1)	365.0 (12.6)	167.0 (12.1)	198.0 (13.1)
Comorbidities						
COPD	1355.0 (31.1)	543.0 (29.9)	812.0 (32.0)	930.0 (32.2)	446.0 (32.4)	484.0 (32.0)
Cancer	676.0 (15.5)	254.0 (14.0)	422.0 (16.6)	465.0 (16.1)	229.0 (16.7)	236.0 (15.6)
Chronic kidney disease	561.0 (12.9)	232.0 (12.8)	329.0 (13.0)	302.0 (10.5)	132.0 (9.6)	170.0 (11.2)

*Note*: Data are reported as *n* (%).

Abbreviations: BMI, body mass index; CAD, coronary artery disease; COPD, chronic obstructive pulmonary disease; SES, socioeconomic status.

**TABLE 2:** Pre‐ and postimplementation cohort characteristics by socioeconomic status

	High SES			Low SES		
Patient characteristics	Total (*N* = 5638)	Pre (*n* = 2501)	Post (*n* = 3137)	Total (*N* = 1607)	Pre (*n* = 692)	Post (*n* = 915)
Age (years), median (IQR)	55 (22)	55 (21)	55 (22)	53 (20)	54 (18)	52 (20)
Female	3027.0 (53.7)	1329.0 (53.1)	1698.0 (54.1)	862.0 (53.6)	365.0 (52.7)	497.0 (54.3)
Race						
White	4257.0 (75.5)	1915.0 (76.6)	2342.0 (74.7)	534.0 (33.2)	224.0 (32.4)	310.0 (33.9)
Non‐White	1381.0 (24.5)	586.0 (23.4)	795.0 (25.3)	1073.0 (66.8)	468.0 (67.6)	605.0 (66.1)
Ethnicity						
Hispanic or Latino	206.0 (3.7)	81.0 (3.2)	125.0 (4.0)	103.0 (6.4)	35.0 (5.1)	68.0 (7.4)
Rural	2539 (45.0)	1213.0 (48.5)	1326.0 (42.3)	348 (21.7)	162.0 (23.4)	186.0 (20.3)
Site						
Academic	4124.0 (73.1)	1779.0 (71.1)	2345.0 (74.8)	1373.0 (85.4)	576.0 (83.2)	797.0 (87.1)
Freestanding	742.0 (13.2)	320.0 (12.8)	422.0 (13.5)	59.0 (3.7)	28.0 (4.0)	31.0 (3.4)
Community	772.0 (13.7)	402.0 (16.1)	370.0 (11.8)	175.0 (10.9)	88.0 (12.7)	87.0 (9.5)
Insurance status						
Private	1736.0 (30.8)	776.0 (31.0)	960.0 (30.6)	267.0 (16.6)	114.0 (16.5)	153.0 (16.7)
Public	2541.0 (45.1)	1068.0 (42.7)	1473.0 (47.0)	846.0 (52.6)	353.0 (51.0)	493.0 (53.9)
Other	1361.0 (24.1)	657.0 (26.3)	704.0 (22.4)	494.0 (30.7)	225.0 (32.5)	269.0 (29.4)
Risk factors						
Prior CAD	1520.0 (27.0)	679.0 (27.1)	841.0 (26.8)	348.0 (21.7)	148.0 (21.4)	200.0 (21.9)
Diabetes	1512.0 (26.8)	691.0 (27.6)	821.0 (26.2)	475.0 (29.6)	202.0 (29.2)	273.0 (29.8)
Hyperlipidemia	2446.0 (43.4)	1058.0 (42.3)	1388.0 (44.2)	616.0 (38.3)	271.0 (39.2)	345.0 (37.7)
Hypertension	3570.0 (63.3)	1612.0 (64.5)	1958.0 (62.4)	1054.0 (65.6)	465.0 (67.2)	589.0 (64.4)
Smoking	3328.0 (59.0)	1520.0 (60.8)	1808.0 (57.6)	1101.0 (68.5)	479.0 (69.2)	622.0 (68.0)
BMI ≥ 30 mg/kg^2^	2527.0 (46.1)	1110.0 (45.4)	1417.0 (46.7)	787.0 (50.6)	342.0 (50.4)	445.0 (50.8)
Cerebrovascular disease	702.0 (12.5)	316.0 (12.6)	386.0 (12.3)	209.0 (13.0)	82.0 (11.8)	127.0 (13.9)
Peripheral vascular disease	744.0 (13.2)	314.0 (12.6)	430.0 (13.7)	205.0 (12.8)	78.0 (11.3)	127.0 (13.9)
Comorbidities						
COPD	1697.0 (30.1)	754.0 (30.1)	943.0 (30.1)	588.0 (36.6)	235.0 (34.0)	353.0 (38.6)
Cancer	952.0 (16.9)	410.0 (16.4)	542.0 (17.3)	189.0 (11.8)	73.0 (10.5)	116.0 (12.7)
Chronic kidney disease	655.0 (11.6)	277.0 (11.1)	378.0 (12.0)	208.0 (12.9)	87.0 (12.6)	121.0 (13.2)

*Note*: Data are reported as *n* (%) unless otherwise specified.

Abbreviations: BMI, body mass index; CAD, coronary artery disease; COPD, chronic obstructive pulmonary disease; SES, socioeconomic status.

**TABLE 3:** HEART pathway test characteristics for the outcome of 30‐day all‐cause death or MI

	Sensitivity (95% CI)	Specificity (95% CI)	PPV (95% CI)	NPV (95% CI)	+LR (95% CI)	−LR (95% CI)
Urban	97.0 (94.4–99.6)	90.9 (89.7–92.3)	42.0 (36.5–47.5)	99.4 (98.9–99.9)	8.80 (7.51–10.32)	0.07 (0.03–0.18)
Rural	99.3 (97.9–100.0)	91.1 (89.4–92.8)	50.8 (43.8–57.7)	99.8 (99.3–100.0)	8.19 (6.61–10.15)	0.02 (0.0–0.13)
Low SES	96.6 (91.9–100.0)	90.1 (87.9–92.3)	38.4 (29.4–47.4)	99.4 (98.5–100.0)	7.50 (5.72–9.83)	0.08 (0.02–0.31)
High SES	98.4 (96.8–100.0)	91.2 (90.1–92.4)	47.4 (42.5–52.3)	99.6 (99.2–100.0)	8.83 (7.63–10.22)	0.04 (0.02–0.11)

Abbreviations: +LR, positive likelihood ratio; –LR, positive likelihood ratio; MI, myocardial infarction; NPV, negative predictive value; PPV, positive predictive value; SES, socioeconomic status.

**TABLE 4:** Safety and effectiveness events by rurality and SES in the pre‐ and postimplementation cohorts

	Urban				Rural				Interaction rurality × implementation cohort *p*‐value
	Total (*N* = 4358)	Pre (*n* = 1818)	Post (*n* = 2540)	aOR^a^ (95% CI)	Total (*N* = 2887)	Pre (*n* = 1375)	Post (*n* = 1512)	aOR^a^ (95% CI)	
Safety									
Index									
Death^b^	10 (0.2)	2 (0.1)	8 (0.3)	2.87 (0.72–19.02)	8 (0.3)	2 (0.1)	6 (0.4)	NA	0.97
MI	246 (5.6)	100 (5.5)	146 (5.7)	1.19 (0.91–1.56)	210 (7.3)	84 (6.1)	126 (8.3)	1.56 (1.16–2.10)*	0.23
Revascularization^c^	130 (3.0)	57 (3.1)	73 (2.9)	1.11 (0.78–1.59)	108 (3.7)	44 (3.2)	64 (4.2)	1.37 (0.92–2.05)	0.29
Death + MI	254 (5.8)	102 (5.6)	152 (6.0)	1.22 (0.93–1.59)	217 (7.5)	86 (6.3)	131 (8.7)	1.59 (1.19–2.14)*	0.24
MACE (death + MI + revascularization)	287 (6.6)	117 (6.4)	170 (6.7)	1.20 (0.93–1.55)	242 (8.4)	103 (7.5)	139 (9.2)	1.37 (1.04–1.81)*	0.51
30 day									
Death^b^	25 (0.6)	8 (0.4)	17 (0.7)	1.52 (0.68–3.74)	27 (0.9)	22 (1.6)	5 (0.3)	0.20 (0.07–0.50)*	0.002*
MI^b^	19 (0.4)	7 (0.4)	12 (0.5)	1.23 (0.49–3.31)	20 (0.7)	9 (0.7)	11 (0.7)	1.11 (0.46–2.77)	0.88
Revascularization^b^	28 (0.6)	7 (0.4)	21 (0.8)	2.16 (0.96–5.49)	32 (1.1)	17 (1.2)	15 (1.0)	0.80 (0.39–1.61)	0.08
Death + MI^d^	41 (0.9)	14 (0.8)	27 (1.1)	1.50 (0.78–2.99)	44 (1.5)	28 (2.0)	16 (1.1)	0.50 (0.26–0.94)*	0.03*
MACE^d^ (death + MI + revascularization)	65 (1.5)	20 (1.1)	45 (1.8)	1.81 (1.07–3.16)	63 (2.2)	40 (2.9)	23 (1.5)	0.53 (0.31–0.89)*	0.002*
30 day (index + follow‐up)									
Death^b^	35 (0.8)	10 (0.6)	25 (1.0)	1.80 (0.89–3.93)	35 (1.2)	24 (1.7)	11 (0.7)	0.41 (0.19–0.83)*	0.005*
MI	253 (5.8)	104 (5.7)	149 (5.9)	1.17 (0.90–1.54)	222 (7.7)	90 (6.5)	132 (8.7)	1.53 (1.15–2.05)*	0.23
Revascularization^e^	157 (3.6)	64 (3.5)	93 (3.7)	1.25 (0.90–1.74)	138 (4.8)	60 (4.4)	78 (5.2)	1.24 (0.88–1.77)	0.82
Death + MI	280 (6.4)	112 (6.2)	168 (6.6)	1.23 (0.95–1.59)	251 (8.7)	110 (8.0)	141 (9.3)	1.30 (0.99–1.71)	0.79
MACE (death + MI + revascularization)	324 (7.4)	129 (7.1)	195 (7.7)	1.26 (0.99–1.61)	281 (9.7)	129 (9.4)	152 (10.1)	1.17 (0.90–1.52)	0.75
Effectiveness									
Index									
Hospitalization	2494 (57.2)	1126 (61.9)	1368 (53.9)	0.75 (0.65–0.86)*	1618 (56.0)	786 (57.2)	832 (55.0)	0.91 (0.77–1.07)	0.03*
OCT	1343 (30.8)	619 (34.0)	724 (28.5)	0.87 (0.75–0.996)*	750 (26.0)	363 (26.4)	387 (25.6)	0.96 (0.80–1.15)	0.27
Early discharge	1763 (40.5)	650 (35.8)	1113 (43.8)	1.36 (1.18–1.57)*	1187 (41.1)	555 (40.4)	632 (41.8)	1.06 (0.90–1.25)	0.01*
30 day (index + follow‐up)									
Hospitalization	2565 (58.9)	1152 (63.4)	1413 (55.6)	0.76 (0.66–0.87)*	1661 (57.5)	815 (59.3)	846 (56.0)	0.86 (0.72–1.02)	0.12
OCT	1488 (34.1)	682 (37.5)	806 (31.7)	0.87 (0.76–0.998)*	863 (29.9)	421 (30.6)	442 (29.2)	0.95 (0.80–1.12)	0.32

*Note*: Data are reported as *n* (%) unless otherwise specified.

Abbreviations: ACS, acute coronary syndrome; aOR, adjusted odds ratio; BMI, body mass index; MACE, major adverse cardiac events; OCT, objective cardiac testing; SES, socioeconomic status.

^a^ Regression models adjusted for rurality (for SES models only), SES (for rurality models only), age, sex, race, BMI, ED location, insurance status, smoking, and presence of chest pain versus other symptoms concerning for ACS.

^b^ Unadjusted due to small number of events.

^c^ Only adjusted for ED location and presence of chest pain versus other symptoms concerning for ACS due to number of events.

^d^ Only adjusted for presence of chest pain versus other symptoms concerning for ACS due to number of events.

^e^ Only adjusted for insurance status, ED location, and presence of chest pain versus other symptoms concerning for ACS due to number of events.

* Statistically significant.

**TABLE 5:** Safety and effectiveness events by rurality and SES in the pre‐ and postimplementation cohorts

	High SES				Low SES				Interaction SES × implementation cohort *p*‐value
	Total (*N* = 5638)	Pre (*n* = 2501)	Post (*n* = 3137)	aOR^a^ (95% CI)	Total (*N* = 1607)	Pre (*n* = 692)	Post (*n* = 915)	aOR^a^ (95% CI)	
Safety									
Index									
Death^b^	15 (0.3)	4 (0.2)	11 (0.4)	2.20 (0.75–7.93)	3 (0.2)	0 (0.0)	3 (0.3)	NA	0.99
MI	363 (6.4)	141 (5.6)	222 (7.1)	1.44 (1.15–1.81)*	93 (5.8)	43 (6.2)	50 (5.5)	1.04 (0.67–1.61)	0.14
Revascularization^c^	202 (3.6)	86 (3.4)	116 (3.7)	1.21 (0.91–1.62)	36 (2.2)	15 (2.2)	21 (2.3)	1.20 (0.61–2.40)	0.97
Death + MI	375 (6.7)	145 (5.8)	230 (7.3)	1.46 (1.17–1.82)*	96 (6.0)	43 (6.2)	53 (5.8)	1.11 (0.72–1.71)	0.19
MACE	424 (7.5)	171 (6.8)	253 (8.1)	1.35 (1.10–1.67)*	105 (6.5)	49 (7.1)	56 (6.1)	1.0 (0.66–1.51)	0.16
30 day									
Death^b^	40 (0.7)	22 (0.9)	18 (0.6)	1.38 (0.67–3.00)	12 (0.7)	8 (1.2)	4 (0.4)	0.38 (0.10–1.20)	0.43
MI^b^	30 (0.5)	11 (0.4)	19 (0.6)	1.20 (0.68–2.15)	9 (0.6)	5 (0.7)	4 (0.4)	NA	0.28
Revascularization^b^	50 (0.9)	20 (0.8)	30 (1.0)	1.20 (0.68–2.15)	10 (0.6)	4 (0.6)	6 (0.7)	1.14 (0.32–4.46)	0.94
Death + MI^d^	64 (1.1)	29 (1.2)	35 (1.1)	0.97 (0.58–1.63)	21 (1.3)	13 (1.9)	8 (0.9)	0.52 (0.20–1.26)	0.16
MACE^d^	100 (1.8)	44 (1.8)	56 (1.8)	1.09 (0.72–1.64)	28 (1.7)	16 (2.3)	12 (1.3)	0.62 (0.28–1.32)	0.18
30 day (index + follow‐up)									
Death^b^	55 (1.0)	26 (1.0)	29 (0.9)	0.89 (0.52–1.52)	15 (0.9)	8 (1.2)	7 (0.8)	0.66 (0.23–1.85)	0.61
MI	376 (6.7)	148 (5.9)	228 (7.3)	1.42 (1.14–1.77)*	99 (6.2)	46 (6.6)	53 (5.8)	1.04 (0.68–1.59)	0.14
Revascularization^e^	250 (4.4)	105 (4.2)	145 (4.6)	1.23 (0.95–1.60)	45 (2.8)	19 (2.7)	26 (2.8)	1.21 (0.66–2.22)	0.85
Death + MI	419 (7.4)	169 (6.8)	250 (8.0)	1.35 (1.09–1.67)*	112 (7.0)	53 (7.7)	59 (6.4)	0.98 (0.66–1.47)	0.11
MACE	483 (8.6)	199 (8.0)	284 (9.1)	1.31 (1.07–1.59)*	122 (7.6)	59 (8.5)	63 (6.9)	0.92 (0.62–1.35)	0.08
Effectiveness									
Index									
Hospitalization	3244 (57.5)	1500 (60.0)	1744 (55.6)	0.84 (0.74–0.94)*	868 (54.0)	412 (59.5)	456 (49.8)	0.72 (0.57–0.90)*	0.20
OCT	1678 (29.8)	786 (31.4)	892 (28.4)	0.90 (0.80–1.02)	415 (25.8)	196 (28.3)	219 (23.9)	0.90 (0.71–1.15)	0.88
Early discharge	2252 (39.9)	943 (37.7)	1309 (41.7)	1.17 (1.04–1.33)*	698 (43.4)	262 (37.9)	436 (47.7)	1.43 (1.14–1.80)*	0.15
30 day (index + follow‐up)									
Hospitalization	3331 (59.1)	1540 (61.6)	1791 (57.1)	0.83 (0.73–0.94)*	895 (55.7)	427 (61.7)	468 (51.1)	0.68 (0.54–0.86)*	0.11
OCT	1896 (33.6)	885 (35.4)	1011 (32.2)	0.90 (0.80–1.02)	455 (28.3)	218 (31.5)	237 (25.9)	0.87 (0.69–1.10)	0.57

*Note*: Data are reported as *n* (%) unless otherwise specified.

Abbreviations: ACS, acute coronary syndrome; aOR, adjusted odds ratio; BMI, body mass index; MACE, major adverse cardiac events; OCT, objective cardiac testing; SES, socioeconomic status.

^a^ Regression models adjusted for rurality (for SES models only), SES (for rurality models only), age, sex, race, BMI, ED location, insurance status, smoking, and presence of chest pain versus other symptoms concerning for ACS.

^b^ Unadjusted due to small number of events.

^c^ Only adjusted for ED location and presence of chest pain versus other symptoms concerning for ACS due to number of events.

^d^ Only adjusted for presence of chest pain versus other symptoms concerning for ACS due to number of events.

^e^ Only adjusted for insurance status, ED location, and presence of chest pain versus other symptoms concerning for ACS due to number of events.

* Statistically significant.

**TABLE 6:** Absolute percentage difference in safety and effectiveness outcomes pre‐ versus postimplementation by rurality and SES

Outcome	Urban % (95% CI)	Rural % (95% CI)	High SES % (95% CI)	Low SES % (95% CI)
Safety				
Index				
Death	0.2 (−0.1 to 0.5)	0.3 (−0.2 to 0.7)	0.2 (−0.1 to 0.5)	0.3 (−0.2 to 0.8)
MI	0.3 (−1.2 to 1.7)	2.2 (0.3 to 4.2)*	1.4 (0.1 to 2.7)*	−0.8 (−3.2 to 1.7)
Revascularization	−0.3 (−1.3 to 0.8)	1.0 (−0.4 to 2.5)	0.3 (−0.8 to 1.3)	0.1 (−1.5 to 1.7)
Death + MI	0.4 (−1.1 to 1.8)	2.4 (0.4 to 4.4)*	1.5 (0.2 to 2.9)*	−0.4 (−2.9 to 2.1)
MACE (death + MI + revascularization)	0.3 (−1.3 to 1.8)	1.7 (−0.4 to 3.8)	1.2 (−0.2 to 2.6)	−1.0 (−3.6 to 1.6)
30‐day follow‐up				
Death	0.2 (−0.3 to 0.7)	−1.3 (−0.5 to −2.1)*	−0.3 (−0.8 to 0.2)	−0.7 (−1.7 to 0.3)
MI	0.1 (−0.4 to 0.5)	0.1 (−0.6 to 0.8)	0.2 (−0.3 to 0.6)	−0.3 (−1.2 to 0.6)
Revascularization	0.4 (−0.1 to 0.9)	−0.2 (−1.1 to 0.6)	0.2 (−0.4 to 0.7)	0.1 (−0.8 to 0.9)
Death + MI	0.3 (−0.3 to 0.9)	−1.0 (−2.0 to 0.0)*	−0.04 (−0.6 to 0.6)	−1.0 (−2.3 to 0.3)
MACE (death + MI + revascularization)	0.7 (−0.1 to 1.4)	−1.4 (−2.5 to −0.2)*	−0.03 (−0.7 to 0.7)	−1.0 (−2.5 to 0.5)
30 day (index + follow‐up)				
Death	0.4 (−0.1 to 1.0)	−1.0 (−1.9 to −0.1)*	−0.1 (−0.7 to 0.4)	−0.4 (−1.5 to 0.7)
MI	0.2 (−1.3 to 1.6)	2.2 (0.2 to 4.2)*	1.4 (0.0 to 2.7)*	−0.9 (−3.4 to 1.7)
Revascularization	0.1 (−1.0 to 1.3)	0.8 (−0.8 to 2.4)	0.4 (0.7 to 1.5)*	0.1 (−1.6 to 1.8)
Death + MI	0.5 (−1.1 to 2.0)	1.3 (−0.8 to 3.4)	1.2 (−0.2 to 2.6)	−1.2 (−3.9 to 1.5)
MACE (death + MI + revascularization)	0.6 (−1.0 to 2.2)	0.7 (−1.6 to 2.9)	1.1 (−0.4 to 2.6)	−1.6 (−4.4 to 1.1)
Effectiveness				
Index				
Hospitalization	−8.1 (−11.1 to −5.1)*	−2.1 (−5.8 to 1.6)	−4.4 (−7.0 to −1.8)*	−9.7 (−14.7 to −4.7)*
OCT	−5.5 (−8.4 to −2.4)*	−0.8 (−4.1 to 2.5)	−3.0 (−5.4 to −0.6)*	−4.4 (−8.9 to 0.1)
Early discharge	8.1 (5.1 to 11.0)*	1.4 (−2.2 to 5.1)	4.0 (1.4 to 6.6)*	9.8 (4.8 to 14.8)*
30 day (index ± follow‐up)				
Hospitalization	−7.7 (−10.7 to −4.8)*	−3.3 (−7.0 to 0.4)	−4.5 (−7.1 to − 1.9)*	−10.6 (−15.5 to −5.6)*
OCT	−5.8 (−8.7 to −2.9)*	−1.4 (−4.8 to 2.0)	−3.2 (−5.7 to − 0.6)*	−5.6 (−10.2 to −1.0)*

Abbreviations: MACE, major adverse cardiac events; OCT, objective cardiac testing; SES, socioeconomic status.

* Statistically significant.

### Rurality

In the postimplementation period, the HEART Pathway identified 28.1% (425/1512) of rural patients as low risk and 53.6% (811/1512) as non–low risk, 6.8% (102/1512) had low‐risk HEAR scores but lacked serial troponin measurements, and 11.5% (174/1512) had an incomplete HEAR score. Among urban patients, 32.2% (818/2540) were low risk and 53.2% (1350/2540) were non–low risk. An additional 7.1% (180/2540) of urban patients were missing serial troponin measurements but had low‐risk HEAR scores, and 7.6% (192/2540) had an incomplete HEAR score. The proportion of rural patients classified as low risk was 4.9% (95% CI 1.1%–7.1%) lower than urban patients (*p* = 0.007).

At 30 days, death or MI occurred in 9.3% (141/1512) of rural patients in the postimplementation cohort compared to 8.0% (110/1375) in the preimplementation cohort (aOR 1.30, 95% CI 0.99–1.77). Among urban patients, death or MI occurred in 6.6% (168/2540) postimplementation versus 6.2% (112/1818) preimplementation (aOR 1.23, 95% CI 0.95–1.59). Among low‐risk patients, 30‐day death or MI occurred in 0.2% (95% CI 0.0%–1.3%) of rural versus 0.6% (95% CI 0.3%–1.4%) urban (*p* = 0.67). The interaction between the HEART Pathway implementation and rural versus urban community was not significant for 30‐day death or MI (*p* = 0.79).

Among rural patients, 56.0% (846/1512) were hospitalized within 30 days in the postimplementation cohort compared to 59.3% (815/1375) preimplementation, a reduction of 3.3% (aOR 0.86, 95% CI 0.72–1.02). In urban patients, 55.6% (1413/2540) were hospitalized postimplementation versus 63.4% (1152/1818) preimplementation, a reduction of 7.8% (aOR 0.76, 95% CI 0.66–0.87). In the postimplementation cohort, nonadherence to the HEART Pathway occurred in 14.4% (178/1236) of rural patients and 15.6% (339/2168) of urban patients (*p* = 0.33).

### SES

In the postimplementation period, use of the HEART Pathway identified 34.0% (311/915) of low SES patients as low risk and 48.9% (447/915) as non–low risk, 7.8% (71/915) had low‐risk HEAR scores but lacked serial troponin measurements, and 9.4% (86/915) had an incomplete HEAR score. Among high SES patients, 29.7% (932/3137) were low risk, 54.6% (1714/3137) non–low risk, 6.7% (211/3137) had low‐risk HEAR scores without serial troponin measurements, and 8.9% (280/3137) had an incomplete HEAR score. The proportion of low SES patients classified as low risk was 4.3% (95% CI 0.7%–7.8%) greater than high SES patients (*p* = 0.02).

Among low SES patients, 30‐day death or MI occurred in 6.4% (59/915) postimplementation versus 7.7% (53/629) preimplementation (aOR 0.98, 95% CI 0.66–1.47). In high SES patients, 30‐day death or MI occurred in 8.0% (250/3137) versus 6.8% (169/2501) in post‐ and preimplementation cohorts, respectively (aOR 1.35, 95% CI 1.09–1.67). However, this was driven by increased detection of index MI events (aOR 1.44, 95% CI 1.15–1.81) and not by death or MI during the 30‐day follow‐up period (aOR 0.97, 95% CI 0.58–1.63). Among low‐risk patients, 30‐day death or MI occurred in 0.6% (95% CI 0.2%–2.3%) of low SES versus 0.4% (95% CI 0.2%–1.1%) of high SES patients (*p* = 0.64). The interaction between the HEART Pathway implementation and low versus high SES was not significant for 30‐day death or MI (*p* = 0.11).

Among patients with low SES, 51.1% (468/915) were hospitalized within 30 days compared to 61.7% (427/692) preimplementation, a reduction of 10.6% (aOR 0.68, 95% CI 0.54–0.86). In high SES patients, 57.1% (1791/3137) were hospitalized postimplementation versus 61.6% (1540/2501) preimplementation, a reduction of 4.5% (aOR 0.83, 95% CI 0.73–0.94). Nonadherence rates were similar in low SES and high SES patients, 14.9% (113/758) versus 15.3% (404/2646; *p* = 0.81), respectively.

## DISCUSSION

This analysis, which is the first to evaluate the performance of the HEART Pathway among rural, urban, low SES, and high SES patients with acute chest pain, demonstrates that clinicians can safely use the HEART Pathway to risk stratify patients in each of these key subgroups. Low‐risk patients, in each subgroup, had 30‐day death or MI rates below the 1% threshold that most providers consider acceptable.^48^ Effectiveness (defined by a 30‐day reduction in hospitalizations) was shown among urban, low SES, and high SES patients, but not in rural patients. Similarly, rates of early discharge were increased by HEART Pathway implementation in all subgroups except rural patients. When adjusting for potential confounders, the HEART Pathway reduced OCT rates only in urban patients.

Given a large body of evidence demonstrating a significant gap in cardiovascular outcomes among rural versus urban patients, the safety of HEART Pathway in rural patients is notable. A recent study by Cross et al.^49^ found that age‐adjusted cardiovascular mortality rates were significantly higher among rural versus urban patients and the mortality gap is widening. In addition, a recent study examining mortality rates among urban and rural hospitals found that death rates for acute MI, acute ischemic stroke, and heart failure were all higher in rural patients.^50^

While safety event rates were similar among rural and urban patients, we did observe a rural disparity in the HEART Pathway effectiveness outcomes. The underlying cause of this difference is unclear and may be multifactorial. We initially hypothesized that providers may have been more likely to admit rural patients, even those at low risk, because of their reduced access to local outpatient resources, such as stress testing, and their distance from tertiary care.

However, adherence to the HEART Pathway disposition recommendations were similar across all subgroups. Thus, physician nonadherence does not appear to explain the effectiveness differences in rural patients. An additional hypothesis is that HEART Pathway effectiveness was reduced, because rural patients have an increased prevalence of cardiovascular risk factors, such as known CAD, hypertension, diabetes, or obesity, in epidemiologic studies.^51^, ^52^, ^53^, ^54^, ^55^ This difference may reduce the proportion of patients classified as low risk and therefore eligible for early discharge from the ED without OCT. In our cohort, rural patients did have a higher prevalence of known CAD. However, rates of other cardiovascular risk factors were similar. Thus, future studies may be needed to explore the cause of HEART Pathway performance differences and how to mitigate this disparity in the rural population.

The HEART Pathway was safe and effective in reducing hospitalizations regardless of SES. There were no significant interactions between HEART Pathway implementation and SES, meaning the effect of implementation on all safety and effectiveness outcomes was not different based on SES. This is important, because prior studies have demonstrated that patients with low SES often suffer meaningful cardiovascular health outcome disparities. A recent study demonstrated that large improvements in cardiovascular mortality have occurred in high SES patients, but not patients with low SES.^56^ Schultz et al.^27^ state that the increased burden of cardiovascular disease in low SES patients is a result of a complicated “constellation of biological, behavioral, and psychosocial risk factors.” However, our study shows that HEART Pathway implementation may standardize chest pain care and achieve similar outcomes for patients regardless of SES.

Prior studies of the HEART Pathway have demonstrated its ability to reduce OCT rates.^13^, ^14^ However, in this analysis, after adjustment for potential confounders, such as age, sex, race, insurance status, enrollment site, smoking, and BMI, the HEART Pathway significantly reduced OCT only in urban patients. Thus, testing rates were similar before and after HEART Pathway implementation in patients regardless of SES and among those from rural areas. These results suggest that providers' heavily weigh other patient factors, beyond their HEART Pathway risk assessments, to determine who receives OCT.

The HEART Pathway has been previously shown to be safe and effective across subgroups of age, sex and race.^14^, ^15^, ^57^, ^58^, ^59^ This study meaningfully adds to the existing HEART Pathway literature by reporting its performance based on rurality and SES. Although studies show that chest pain accounts for 3.5% of rural ED visits, no prior analyses have evaluated the performance of a chest pain ADP specifically in rural patient populations.^60^ Furthermore, while recent data suggest that patients with a low SES have high incidence of chest pain and experience disparities in chest pain care and outcomes, no prior studies have focused on ADP performance in patients with low SES.^28^ Thus, this study is an important step in the investigation of chest pain related health care disparities. With this new evidence in hand, emergency clinicians can confidently use the HEART Pathway in their everyday practice. Clinicians can safely risk stratify patients, regardless of SES. However, clinicians must also be aware that the HEART Pathway is associated with decreased hospitalization and increased early discharges among urban, but not rural, patients. Future studies should focus on equalizing care for patients living in rural areas and for those with low SES. Based on our results, broad implementation of the HEART Pathway may reduce disparities in chest pain care for patients with low SES, but is unlikely to reduce rural disparities.

## LIMITATIONS

This study has limitations. Although our three sites were diverse in size and location, the results may not be generalizable to all health systems. Secular trends and provider maturation effects are possible threats to the validity of the results. However, event rates in the study were consistent over time. The HEART Pathway Implementation Study was completed 6 years ago, before high‐sensitivity troponin assays were available in the United States. However, despite the age of the data, our results provide new insights about the performance of the HEART Pathway, which remains relevant as it is still widely used for chest pain risk stratification in U.S. EDs. Using the EHR to collect events may have decreased event detection compared to traditional methods of follow‐up. However, supplementing the EHR with death index and claims data identified only 16 additional 30‐day safety events. Finally, SES was determined based on patients' neighborhood census tract instead of their income. However, the use of ADI‐derived SES is consistent with prior studies and is strongly correlated with other individual SES measures.^37^, ^39^ In this study, high versus low SES was determined using the 85th ADI percentile. This is consistent with the original ADI methodology literature and supported by the Centers for Disease Control and Prevention.^39^, ^41^, ^42^ However, other methods involving quintiles, quartiles, and the 50th ADI percentile exist.^61^, ^62^ Some patients were missing zip code and/or GEOID data. These missing data were due to addresses being incomplete or erroneous or there being a mismatch between address, city, or state. Patients experiencing homelessness were not systematically excluded.

## CONCLUSIONS

Clinicians can safety use the HEART Pathway to risk stratify patients with acute chest pain regardless of their rurality or socioeconomic status. Effectiveness of the HEART Pathway in decreasing hospitalizations and increasing early discharges was demonstrated in urban, low socioeconomic status, and high socioeconomic status groups, but not rural patients. Reductions in OCT rates were only seen in urban patients. Further study is needed to determine why the effectiveness of the HEART Pathway differed in the rural population.

## FUNDING INFORMATION

This project was funded by the Donaghue Foundation and the Association of American Medical Colleges (AAMC). Dr. O'Neill receives funding from Cytovale. Dr. Ashburn receives funding from NHLBI (T32HL076132). The content is solely the responsibility of the authors and does not necessarily represent the official views of the NIH. Dr. Snavely receives funding from Abbott Laboratories and HRSA (1H2ARH399760100). Dr. Stopyra receives research funding from NCATS/NIH (KL2TR001421), HRSA (H2ARH39976‐01‐00), Roche Diagnostics, Abbott Laboratories, Pathfast, Genetesis, Cytovale, Forest Devices, Vifor Pharma, and Chiesi Farmaceutici. Dr. Mahler receives funding/support from Roche Diagnostics, Abbott Laboratories, Ortho Clinical Diagnostics, Siemens, Grifols, Pathfast, Quidel, Genetesis, Cytovale, and HRSA (1H2ARH399760100). He is a consultant for Roche, Quidel, Genetesis, Inflammatix, Radiometer, and Amgen and the chief medical officer for Impathiq Inc.

## CONFLICT OF INTEREST

The authors declare no potential conflict of interest.

## Supporting information


Table S1

Figure S1
