# Supplementary material for: Rural and socioeconomic differences in the effectiveness of the HEART Pathway accelerated diagnostic protocol
Source: Acad Emerg Med. 2023 Jan 3;30(2):110–23. doi: 10.1111/acem.14643 (PMC10009897; doi:10.1111/acem.14643)
Supplement: Supplementary file 1 — Table S1 [file ACEM-30-110-s002.docx]

**Supplemental Table 1. Safety events at 30-days among low-risk patients presented by group**

|  | **Urban/ Rural** | **SES high/low** | **Age (Years)** | **Sex** | **Race** | **Comorbidities** | **Site** | **HEAR Score** | **Event** |
| --- | --- | --- | --- | --- | --- | --- | --- | --- | --- |
| **Patient #1** | **Urban** | **Low** | **57** | **Female** | **Black** | **Metastatic uterine cancer deep vein thrombosis on enoxaparin.** | **Academic** | **3** | **Death during index hospitalization; care withdrawn** |
| **Patient #2** | **Urban** | **Low** | **73** | **Male** | **White** | **Chronic obstructive pulmonary disease** | **Academic** | **2** | **Returned to the ED and died 6 days after index from subarachnoid hemorrhage** |
| **Patient #3** | **Urban** | **High** | **41** | **Female** | **White** | **Hypertension, Hyperlipidemia, Diabetes, Obesity, Family history of ACS** | **Freestanding** | **3** | **Index visit non-ST-segment elevation myocardial infarction with referral for CABG** |
| **Patient #4** | **Urban** | **High** | **43** | **Male** | **White** | **None** | **Academic** | **0** | **Death during index visit respiratory failure and pulseless electric activity arrest** |
| **Patient #5** | **Urban** | **High** | **50** | **Male** | **Black** | **Hypertension tobacco cocaine use** | **Academic** | **3** | **Returned to ED 12 days after index visit with STEMI** |
| **Patient #6** | **Rural** | **High** | **76** | **Female** | **Black** | **Hypertension autoimmune hepatitis** | **Academic** | **3** | **Death on day 28; admitted to outside hospital for acute encephalopathy** |
