# Supplementary figures and images for: Rural and socioeconomic differences in the effectiveness of the HEART Pathway accelerated diagnostic protocol
Source: Acad Emerg Med. 2023 Jan 3;30(2):110–23. doi: 10.1111/acem.14643 (PMC10009897; doi:10.1111/acem.14643)

**Supplemental Figure 1. Patient flow diagram**

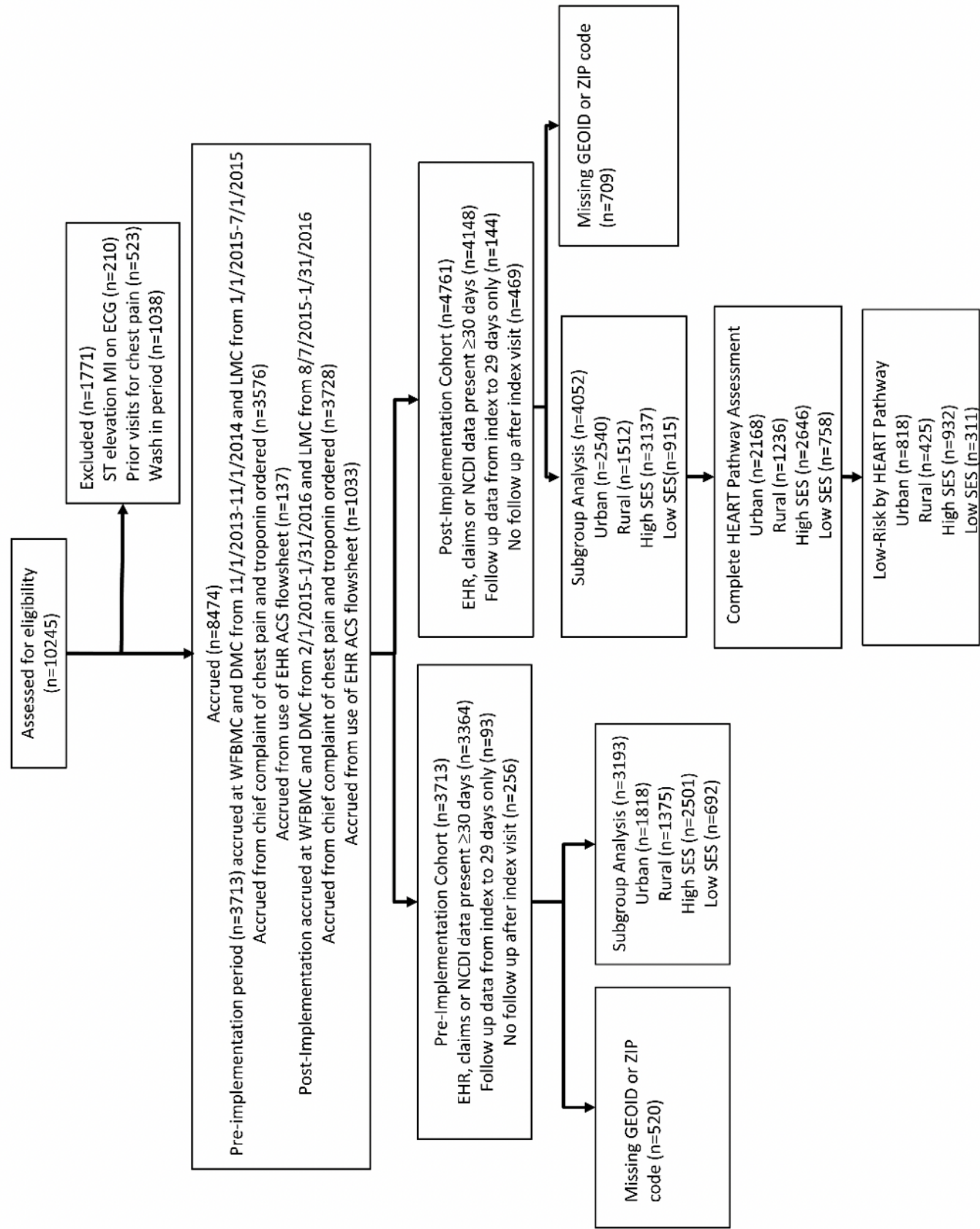

Supplement: Supplementary file 2 — Figure S1 [file ACEM-30-110-s001.pdf]
